# Association between Dental Caries and Handgrip Strength: In a Population-Based Study in Korea (KNHANES 2016–2018)

**DOI:** 10.3390/ijerph19169874

**Published:** 2022-08-10

**Authors:** Eun-Jeong Kim, Chae-Hee Lim, Min Eun, Su-A Yu, So-Min Kwon, Jeong-Eun Lee, Kyu-Ri Lee, Se-Hyun Park, Hye-Ju Lee

**Affiliations:** 1 Department of Dental Hygiene, Gangdong University, Eumseong-gun 27600, Chungcheongbuk-do, Korea; 2 Department of Dental Hygiene, College of Health Science, Sun Moon University, Asan-si 31460, Chungcheongnam-do, Korea

**Keywords:** dental caries, handgrip strength, oral health, KNHANES

## Abstract

This study aimed to verify the relationship between handgrip strength and oral health using data from the Korea National Health and Nutrition Examination Survey, representing Korean adults. Data from the seventh survey (2016–2018) conducted by the Korea Centers for Disease Control and Prevention were utilized, and 10,607 final study participants were recruited according to the selection and exclusion criteria. A complex sample logistic regression analysis was performed to confirm the relationship between handgrip strength and dental caries according to the sex of the study participants. On analyzing the correlation in men, “C1 (DMFT: 11–32),” when compared to “C4 (DMFT: 0–3),” in Model 1 without adjustment for potential confounders, was 2.92 (95% confidence interval [CI]: 2.15–3.97) times more likely to be associated with lower handgrip strength, and a statistically significant result was detected (*p* < 0.001). Additionally, significant odds ratios (ORs) were confirmed for all adjusted models. In women, the ORs in Model 1 without adjustment for potential confounders were 1.41 times (95% CI: 1.14–1.75) and demonstrated a significant result; however, the results were not significant in all adjusted models 2–4. Resultantly, a significant association was detected between dental caries and handgrip strength in Korean adults.

## 1. Introduction

Modern individuals’ interest in health is persistently increasing owing to the improvement in education level and economic leeway, and several studies related to health have been reported [1,2,3]. Systemic diseases, including chronic diseases, frequently occur in individuals [4] and are becoming increasingly important for general health.

Particularly, general health and various areas of life are associated with handgrip strength. Handgrip strength is an index that predicts muscle strength and is an important biomarker of disease and health [2]. The motor functions of the hand are classified into manipulation, support, and prehension [5]. Particularly, handgrip force, which indicates the power to hand-grip an object, consists of the coordination of the four fingers and thumb [6]. Handgrip strength reportedly decreases with age, causing discomfort in daily life [1].

According to previous studies, low handgrip strength is related to various chronic diseases, including cardiovascular disease and diabetes mellitus. In another study, the mortality rate was 79% higher in the group with low handgrip strength than that in the group with high handgrip strength, and when the handgrip strength increased by 1 kg, the mortality rate was reduced by 4% [7]. Additionally, handgrip strength is reportedly not only associated with the prevalence of hypertension and circulatory disease [3], but also with health-related quality of life [8]. Thus, it was possible to confirm the relationship between handgrip strength and general health in previous studies [2,9,10].

Because oral health is closely associated with systemic diseases and substantially influences physical health [11,12,13], it is critical to maintain oral health. In particular, treatment and prevention of dental caries, a representative oral disease, is essential [11,12]. Thorough management of dental plaque is critical to maintain oral health [14]. Moreover, physical and chemical dental biofilm management can prevent the adhesion and accumulation of dental plaque [15]. Tooth brushing is recommended as the most effective and convenient method for managing biofilms on teeth [14,15,16]. According to the brushing force and frequency, a significant difference in the occurrence of caries has been noted. Moreover, there is a difference in the occurrence of periodontal diseases and gingivitis depending on the brushing method, time, force, and frequency [17]. As brushing requires coordination between hand and arm movements, handgrip strength can be an important physical factor in oral hygiene management [18]. According to research, handgrip strength not only directly or indirectly affects the brushing effect but is also related to the accumulation and degree of dental plaque [14]. Decreased hand agility and handgrip strength are factors that increase the maturation of dental plaque and are reportedly related to oral hygiene [19,20].

Although studies on the relationship between handgrip strength and periodontal disease have been reported [21], those evaluating the relationship between dental caries and handgrip strength are scarce. Therefore, this study aimed to use data from the National Health and Nutrition Examination Survey to verify the relationship between dental caries and handgrip strength in Korean adults.

## 2. Materials and Methods

### 2.1. Study Subjects and Data Collection

The dataset used in this study, KNHANES (2016–2018), is an annual survey conducted by the Centers for Disease Control and Prevention and the Ministry of Health and Welfare to monitor the general health and nutritional status of the people. The Institutional Review Board (IRB) of the Centers for Disease Control and Prevention has approved this national investigation (IRB number: 2018-01-03-P-A). Trained interviewers and examiners collected data from study participants through health interviews, health screenings, and nutritional surveys. All research procedures were conducted in accordance with the Declaration of Helsinki. In this study, data from 10,607 persons, excluding missing values among 16,489 respondents, were used.

### 2.2. Handgrip Strength

A digital handgrip strength dynamometer (T.K.K 5401, Japan) was used to measure the maximum force. Measurements were performed after the legs were spread to the width of the pelvis, the front region was observed, and the hips were raised straight such that they prevented the arms from reaching the body. While maintaining the patient’s basic posture, the dynamometer was squeezed with the thumb and four fingers, and the patient was instructed to pull the handgrip forcefully for 3 s. Handgrip strength was measured thrice alternately with both hands, and the average of the three values of the right handgrip strength was used. Based on the “Asian working group for sarcopenia,” the measured values were reclassified into above-average (men: 26 kg or more, women: 18 kg or more) and below-average groups (men: less than 26 kg, women: less than 18 kg) [22].

### 2.3. Dental Caries

A trained dentist examined dental caries using a mouth mirror under fluorescent lighting. This assessment was performed using the method proposed by the World Health Organization [20], and the DMFT scores were classified into four groups: C1 (11–32), C2 (7–1), C3 (4–6), and C4 (0–3).

### 2.4. Assessment of Confounders

Sex and age, constitution index (body mass index (BMI)), smoking, education level, drinking, marital status, physical activity, hypertension, diabetes, economic level, brushing frequency, interdental toothbrush use, and electric toothbrush use were assessed using the National Health and Nutrition Examination Health Questionnaire Survey. The presence or absence of dental visits and oral examinations for 1 year were also noted.

Based on age, the participants were divided into five groups: “20–29”, “30–39”, “40–49”, “50–59”, and “≥60 years.” Obesity was categorized as “yes” if BMI was 25 kg/m^2^ or higher and “no” when BMI was lower than 25 kg/m^2^. Education levels were categorized as “less than elementary school graduates,” “middle school graduates,” “high school graduates,” and “more than college graduates.” Smoking was classified into “non-smokers”, “small smokers (less than 10 cigarettes a day),” and “heavy smokers (≥10 cigarettes a day)” with reference to previous studies [23]. Participants were divided into “drinkers (≧1 cup)” and “non-drinkers (0 cups)” based on whether they consumed alcohol. The participants’ marital status was classified as “married” or “unmarried,” and physical activity as “inactive” or “active.” The presence or absence of hypertension and diabetes was categorized as “yes” or “no” depending on whether or not it was diagnosed by a physician. Quartile household income was divided into the “lower,” “ middle-lower,” “middle-upper,” and “upper” groups. Brushing frequency was divided into “one time or more,” “two times,” and “three times or more,” and the possibility of a 1-year dental visit was divided into “done” and “did not”. Whether oral examination was possible was divided into “done” and “not done.” The participants’ use of an electric toothbrush was divided into “used” and “not used,” and the presence or absence of interdental toothbrush use was also divided into “used” and “not used.”

### 2.5. Data Analysis

IBM SPSS ver. 25.0 (IBM Co., Armonk, NY, USA) was used for the analyses. In this study, a composite sample cross-analysis was performed to understand the general characteristics of the study participants and the distribution of handgrip strength due to dental caries. Moreover, composite sample logistic regression was performed to confirm the relationship between handgrip strength and dental caries.

## 3. Results

### 3.1. General Characteristics

The general characteristics of the study participants were the same as those in Table 1. Of the 10,607 participants, 4691 were men and 5916 were women, with an average age of 49.9 years. In terms of age, 60 years and older were the most common (*p* < 0.001). The income quartile tended to be lower in the order of middle-upper, upper, middle-lower, and lower; however, no statistically significant difference was detected. The education level was highest for university graduates and gradually decreased in the order of high, junior high, and elementary school education (*p* < 0.001). Regarding marital status, there were more married than unmarried (*p* < 0.001). There were more non-smokers than smokers and more drinkers than non-drinkers (*p* < 0.001). Regarding the physical activity state, there were more inactive people than active people (*p* < 0.001). The prevalence was lower in patients with and without a diagnosis of hypertension or diabetes (*p* < 0.001). There were also significantly more people of normal weight than those who were obese (*p* < 0.001). There was also a significant difference in the survey on oral examination and interdental toothbrush use.

### 3.2. Dental Caries and Handgrip Strength by Sex

The dependent variable was handgrip strength (high/low), and the independent variable was dental caries (four groups of permanent teeth with caries experience). As shown in Table 2, the proportion of low handgrip strength increased as the f DMFT scores increased for both men and women, demonstrating a significant difference (*p* < 0.001).

### 3.3. Relationship between Dental Caries and Handgrip Strength by Sex

Table 3 shows the relationship between dental caries and handgrip strength according to sex. It was 2.92 (2.15–3.97) times more likely that men had lower handgrip strength in “C1” than that of “C4” in Model 1 with no adjustments, with the results being significant (*p* < 0.001). In the age-adjusted Model 2, the possibility that the handgrip strength of “C1” was lower than that of “C4” was 2.15 (1.56–2.96) times higher, and statistically significant results were obtained. Model 3, adjusted for age, income quartile education level, and marital status, was 1.79 (1.29–2.47) times more likely to have less handgrip strength in “C1” than that of “C4,” confirming statistically significant results (*p* < 0.001). Model 4 was adjusted for age, income quartile, education level, marital status, hypertension, diabetes, smoking, drinking frequency, and physical activity, and the possibility that “C1” is associated with lower handgrip strength than that of “C4” was 1.62 (1.17–2.26) times higher, and the result was statistically significant (*p* = 0.002). Model 5, adjusted for all variables, was 1.61 (1.15–2.26) times more likely to have less handgrip strength in “C1” than “C4.” This showed statistically significant results (*p* = 0.003). In women, the OR in Model 1 without adjusting for the variables showed a significant result at 1.41 (1.14–1.75) times; however, this difference was not significant in corrected Models 2–4.

## 4. Discussion

This study aimed to demonstrate the relationship between dental caries and handgrip strength. As hypothesized in this study that handgrip strength is associated with the caries of permanent teeth, men demonstrated significance for all models, and women did not exhibit significant results for any models, except Model 1.There are previous studies showing differences by gender in oral health-related indicators: the number of daily brushings and degree of interest in caries prevention is reportedly higher in women than in men, and oral supplement use was also higher in women than in men [24]. According to a previous study that analyzed handgrip strength according to gender, it was confirmed that the older group had stronger grip strength. This is because women in their 20s who participated in the study were mainly white-collar workers who did not engage in physical labor or muscular activity, those aged in their 30s and 40s were primarily housewives and those in their 50s were mainly field workers. However, in the case of men, all age groups were engaged in production jobs, and the results of the study showed that muscle strength decreased with increasing age. For this reason, differences in grip strength according to age and gender were confirmed. [25]. According to previous studies on oral health management by sex, women were more active than men in oral health management and prevention, such as the number of times of tooth brushing and interest in caries prevention according to sex. Women who care about their appearance are more active in oral health care and prevention than men as they value esthetics. This reveals differences in maintaining and promoting oral health according to sex [24,26]. 

There were few studies on this subject, and previous studies examined the relationship between handgrip strength and other oral health indicators. When the difference between oral health and handgrip strength is divided by sex, remaining teeth and handgrip strength are related in men; however, periodontal disease and handgrip strength are unrelated, and in women, no correlation was observed between the oral index and handgrip strength. This is because the reason for extraction differed depending on sex. Many women were offered dentures for esthetic reasons at an early age when their teeth were extracted. For men, the number of remaining teeth was likely an alternative measure of the degree of lifetime exposure to inflammation that had a detrimental effect on muscle mass and strength over time [27]. This result is similar to the sex-dependent difference in significance probability observed in this study. We also confirmed the results of previous studies showing that brushing force is not related to the removal of bacterial membranes on the tooth surface [28].

Previous studies examining the association between handgrip strength and oral indicators have shown that low handgrip strength promotes premature hand fatigue, which leads to reduced brushing time and severe periodontal disease. Poor oral hygiene increases the likelihood of periodontal diseases. This proves that low handgrip strength is a risk factor contributing to the development of periodontal disease [14]. According to Vinish Aravindakshan, poor oral health and periodontal disease are associated with low handgrip strength. Additionally, the significant relationship between periodontitis and handgrip strength persisted after adjusting for age and sex, but not for the remaining variables [21]. We speculate that these results differed because the activity of dental caries decreases as age increases, but periodontal disease further increases. Additionally, a previous study showed that elderly people with reduced hand agility and handgrip strength have a reduced oral health behavior utilization rate (brushing) and are vulnerable to poor oral health [29]. Previous research on the relationship between grip strength and oral health supports our hypothesis and results. Plaque removal is essential to prevent tooth decay and periodontal disease, the major causes of tooth loss [30]. Plaque removal is performed by hand and arm movements, and it is difficult to physically remove plaque because the decrease in grip strength affects not only brushing teeth but also the patient’s ability to perform oral care such as interdental brushing [31]. Therefore, low handgrip strength can also affect dental caries. According to previous studies, decreased handgrip strength was associated with disability, sarcopenia, and limited dexterity [31,32], and another study reported a relationship between limited or inadequate dexterity and poor oral care [33]. However, handgrip strength cannot fully explain the prevalence of dental caries. This is because various oral health behaviors such as the use of oral hygiene products and medical care can become potential risk factors for dental caries. In Model 5 of Table 3, we analyzed whether potential confounding factors such as frequency and method of brushing, use of oral hygiene products such as interdental and electric toothbrushes, dental clinic visits, and oral examinations had additional effects on the relationship between handgrip strength and dental caries. Compared with Model 4, excluding these confounding factors, their effects could hardly be confirmed. Therefore, a more well-designed further study to comprehensively analyze the prevalence of dental caries according to these potential confounding factors will be needed. The disadvantages of this study are as follows: First, this is a cross-sectional study that can confirm the relationship between handgrip strength and dental caries but cannot explain a clear causal relationship between them. In addition, when analyzing the relationship between handgrip strength and dental caries, a detailed analysis of potential risk factors such as the frequency and method of oral hygiene product use and dental clinic visits was not performed. Further study on this will be needed. Despite these limitations, the advantages of this study are as follows: First, unlike previous studies that used residual teeth, dental prostheses, and periodontal disease as oral health indicators to examine the relationship between handgrip strength and oral health, this study used a tooth decay index to examine the relationship between oral health and handgrip strength. This is also the first study to confirm the relationship between handgrip strength and dental caries using data from a large population of Korean adults based on the National Health and Nutrition Examination Big Data.

## 5. Conclusions

A significant association was detected between dental caries and handgrip strength in Korean adults, and a follow-up study will be needed to clarify the causal relationship between them.

## Figures and Tables

**Table 1:** General characteristics according to sex.

Variables		Total (N)	Male	Female	*p*-Value *
			*N* (%)	*N* (%)	
Age					
	20–29	1316	643 (15.0)	673 (11.4)	**<0.001**
	30–39	1661	736 (15.6)	925 (15.3)	
	40–49	2017	854 (16.7)	1163 (19.0)	
	50–59	2059	853 (18.4)	1206 (22.5)	
	60 over	3554	1605 (34.3)	1949 (31.9)	
Income (4th quartile)					
	Lowest quartile	1812	766 (16.1)	1046 (17.3)	0.142
	Lower middle quartile	2661	1148 (24.1)	1513 (25.1)	
	Upper middle quartile	3082	1379 (29.6)	1703 (28.6)	
	Highest quartile	3052	1398 (30.2)	1654 (29.0)	
Educational level					
	Primary school	1548	489 (9.9)	1059 (17.0)	**<0.001**
	Middle school	1193	515 (11.0)	678 (11.5)	
	High school	3120	1376 (28.9)	1744 (30.7)	
	College	4746	2311 (50.2)	2435 (40.8)	
Married status					
	Yes	8685	3618 (75.6)	5067 (85.7)	**<0.001**
	No	1922	1073 (24.4)	849 (14.3)	
Smoking status					
	None	8642	3028 (65.1)	5614 (94.6)	**<0.001**
	Small smoker	480	314 (6.8)	166 (3.1)	
	Heavy smoker	1485	1349 (28.1)	136 (2.3)	
Drinking status					
	Yes	7878	3922 (83.6)	3956 (67.8)	**<0.001**
	No	2729	769 (16.4)	1960 (32.2)	
Physical activity					
	Inactive	9533	4009 (84.9)	5524 (93.0)	**<0.001**
	Active	1074	682 (15.1)	392 (7.0)	
Hypertension					
	Yes	2340	1153 (24.8)	1187 (19.0)	**<0.001**
	No	8267	3538 (75.2)	4729 (81.0)	
Diabetes					
	Yes	951	496 (10.4)	455 (7.3)	**<0.001**
	No	9656	4195 (89.6)	5461 (92.7)	
Obesity					
	Yes	3644	1892 (39.7)	1752 (29.0)	**<0.001**
	No	6963	2799 (60.3)	4164 (71.0)	
Daily tooth brushing					
	1≧	2948	1548 (32.9)	1400 (23.8)	**<0.001**
	2	3903	1678 (35.3)	2225 (37.7)	
	3≦	3756	1465 (31.8)	2291 (38.5)	
Dental clinic visit					
	Yes	6169	2703 (57.2)	3466 (58.7)	0.142
	No	4438	1988 (42.8)	2450 (41.3)	
Oral examination					
	Yes	3837	1621 (34.4)	2216 (37.8)	**0.002**
	No	6770	3070 (65.6)	3700 (62.2)	
Use of electric toothbrush					
	Yes	497	236 (4.8)	261 (4.8)	0.888
	No	10,110	4455 (95.2)	5655 (95.2)	
Use of interdental brush					
	Yes	2007	757 (16.2)	1250 (20.9)	**<0.001**
	No	8600	3934 (83.8)	4666 (79.1)	

* Obtained from chi-square test. Bold type denotes statistical significance at *p*-value < 0.05.

**Table 2:** Handgrip strength and dental caries according to sex.

Variables		Male		*p*-Value *	Female		*p*-Value *
		*N* (%)	*N* (%)		*N* (%)	*N* (%)	
DMFT		Reduction ^a^	Normal		Reduction ^a^	Normal	
	0–3	101 (23.3)	1339 (31.9)	**<0.001**	255 (19.0)	943 (21.2)	**<0.001**
	4–6	73 (18.6)	940 (22.0)		251 (19.5)	961 (20.5)	
	7–10	71 (15.5)	1082 (26.1)		362 (26.5)	1396 (30.6)	
	11–32	176 (42.6)	909 (20.0)		481 (35.0)	1267 (27.7)	

* Obtained from chi-square test. Bold type denotes statistical significance at *p*-value < 0.05. ^a^ Cut-off value of handgrip strength (<26 kg in males and <18 kg in females).

**Table 3:** Association between dental caries and handgrip strength according to sex.

Model	Male			Female		
	OR	95% CI	*p*-Value *	OR	95% CI	*p*-Value *
Model1 ^a^	2.92	2.15 ~ 3.97	**<0.001**	1.41	1.14 ~ 1.75	**<0.001**
Model2 ^b^	2.15	1.56 ~ 2.96	**<0.001**	1.22	0.98 ~ 1.52	0.129
Model3 ^c^	1.79	1.29 ~ 2.47	**<0.001**	1.18	0.94 ~ 1.47	0.387
Model4 ^d^	1.62	1.17 ~ 2.26	**0.002**	1.16	0.93 ~ 1.46	0.452
Model5 ^e^	1.61	1.15 ~ 2.26	**0.003**	1.16	0.93 ~ 1.45	0.481
Dependent variable: handgrip strength (reference: normal).						
Data are presented as 95% confidence intervals.						

* Obtained from Logistic regression. OR: odds ratio, ^a^ Model1: consisted of a crude association, ^b^ Model2: adjusted for age, ^c^ Model3: adjusted for age, income, educational level, married status, ^d^ Model4: adjusted for age, income, educational level, married status, hypertension, diabetes, smoking, alcohol frequency, obesity, physical activity, ^e^ Model5: adjusted for age, income, educational level, married status, hypertension, diabetes, smoking, alcohol frequency, obesity, physical activity, dental clinic visit, oral examination, daily tooth brushing, use of electric toothbrush, use of interdental brush. Bold values denotes statistical significance at *p* < 0.05.

## Data Availability

The data from the KNHANES VII survey can be accessed and downloaded from the KNHANES homepage (URL: https://knhanes.kdca.go.kr/knhanes/sub03/sub03_02_05.do, accessed on 24 January 2022).

## References

[1] Lee K.S., Woo K.J., Shim J.H., Lee G.H. (1995). The clinical study of grip and pinch strength in normal korean adult. J. Korean Orthop. Assoc..

[2] Bohannon R.W. (2015). Muscle strength clinical and prognostic value of hand-grip dynamometry. Curr. Opin. Clin. Nutr. Metab. Care.

[3] Lee J.A. (2017). Relationship between grip strength and prevalence of hypertension in Korean adults: The sixth Korea national health and nutrition examination survey. Off. J. Korean Acad. Kinesiol..

[4] Kim C.S., Choi Y.K. (2017). Survey of Adults’ Perceptions of the Association between Chronic Diseases and Oral Health. J. Dent. Hyg. Sci..

[5] Neumann D. (2009). Kinesiology of the Musculoskeletal System: Foundations for Rehabilitation.

[6] Massy-Westropp N.M., Gill T.K., Taylor A.W., Bohannon R.W., Hill C.L. (2001). Hand grip strength: Age and gender stratified normative data in a population-based study. BMC Res. Notes.

[7] Rijk J.M., Roos P.R., Deckx L., van den Akker M., Buntinx F. (2016). Prognostic value of handgrip strength in people aged 60 years and older: A systematic review and meta-analysis. Geriatr. Gerontol. Int..

[8] Oh Y.H., Moon J.H., Kong M.H., Oh B.J., Kim H.J. (2017). The Association between Hand Grip Strength and Health-Related Quality of Life in Korean Adults. Korean J. Sports Med..

[9] Hong S.H., Byeon J.Y., Min J.H., Park D.H., Cho W.H., Jeon Y.G. (2021). Relationship Between Handgrip Strength and the Prevalence of Diabetes Mellitus Among Korean Adults: Korean National Health and Nutrition Examination Survey, 2014–2018. Exerc. Sci..

[10] Cho J.K., Yoon Y.S., Park S.H. (2019). Association of Relative Handgrip Strength with the Incidence of Metabolic Syndrome in Korean Adults: A Community Based Cohort Study. Exerc. Sci..

[11] Jung M.H., Kim J.Y. (2007). Community periodontal index treatment needs in relation to dental health care of migrant worker. J. Korean Soc. Dent. Hyg..

[12] Kim J.Y., Jung M.H. (2007). A study on oral health state of immigrant workers with DMFT-index. J Korean Soc. Dent. Hyg..

[13] Shin H.S., Ahn Y.S., Lim D.S. (2016). Association between the number of existing permanent teeth and chronic obstructive pulmonary disease. J. Dent. Hyg. Sci..

[14] An H.R. (2020). The Association between Handgrip Strength and Periodontal Disease in Korean Adults Aged 30 Years and Older: Data from the Korean National Health and Nutrition Examination Survey 2014, 2015. Master’s Thesis.

[15] Kim H.N., Kim M.J., Kim J.E. (2013). Effect of gingival health promotion and oral hygiene improvement for children using vibratory toothbrushes. J. Korean Acad. Oral Health.

[16] Kim Y.K., Han M.D. (2003). Oral Microbiology.

[17] Bae S.M., Kim H.J. (2015). Oral health status of the cognitive and behavioral brushing oral health problems in some adults. AJMAHS.

[18] Yang B.I., Park J.A., Lee J.Y., Jin B.H. (2021). A study on the influencing factors of oral health-related behaviors of the elderly in elderly welfare facilities on oral health-relatd quality of life. J. Korean Acad. Oral Health.

[19] Shin N.R. (2018). Factors Influencing Dental Plaque Maturation in Older Korean: Focusing on Hand Function. Master’s Thesis.

[20] Yusuke M., Ken-ichi M., Kazunori I. (2018). Association of handgrip strength with various oral functions in 82 to 84-year old community dwelling Japanese. Gerodontology.

[21] Aravindakshan V., Hakeem F.F., Sabbah W. (2020). Periodontal disease and grip strength among older adults. Geriatrics.

[22] Chen L.K., Liu L.K., Woo J., Assantachai P., Auyeung T.W., Bahyah K.S., Chou M.Y., Chen L.Y., Hsu P.S., Krairit O. (2014). Sarcopenia in asia: Consensus report of the Asian working group for sarcopenia. J. Am. Med. Dir. Assoc..

[23] Luciano A., Bolognani M., Biondani P., Ghizzi C., Zoppi G., Signori E. (1998). The influence of maternal passive and light active smoking on intrauterine growth and body composition of the newborn. Eur. J. Clin. Nutr..

[24] Jin H.J., Jung E.K., Lee Y.E., Song K.B. (2015). Cognition of dental caries prevention by the level of the social economic status in Korea: Based on Gallup survey. J. Korean Soc. Dent. Hyg..

[25] Lee D.C., Jang K.P. (1997). An Analysis of Grip Strength for Korean Adults. JESK.

[26] Song I.S., Han K., Choi Y.J., Ryu J.J., Park J.B. (2016). Influence of oral health behavior and socio demographic factors on remaining teeth in Korean adults: 2010–2012 Korea national health and nutrition examination survey. Medicine.

[27] Hämäläinen P., Rantanen T., Keskinen M., Meurman J.H. (2004). Oral health status and change in handgrip strength over a 5-year period in 80-year-old people. Gerodontology.

[28] Van der Weijden G.A., Timmerman M.F., Danser M.M., Van der Velden U. (1998). Relationship between the plaque removal efficacy of a manual toothbrush and brushing force. J. Clin. Periodontol..

[29] Yun J.H., Lee Y.H. (2020). Association between oral health status and handgrip strength in older Korean adults. Eur. Geriatr. Med..

[30] Han K., Park J.B. (2017). Association between oral health behavior and periodontal disease among Korean adults: The Korea national health and nutrition examination survey. Medicine.

[31] Felder R., James K., Brown C., Lemon S., Reveal M. (1994). Dexterity testing as a predictor of oral care ability. J. Am. Geriatr. Soc..

[32] Tanimoto Y., Watanabe M., Sun W., Sugiura Y., Tsuda Y., Kimura M., Hayashida I., Kusabiraki T., Kono K. (2012). Association between sarcopenia and higher-level functional capacity in daily living in community-dwelling elderly subjects in Japan. Arch. Gerontol. Geriatr..

[33] Kim C.R., Jeon Y.J., Jeong T. (2019). Risk factors associated with low handgrip strength in the older Korean population. PLoS ONE.

